# Dietary intake and eating behavior in depot medroxyprogesterone acetate users: a systematic review

**DOI:** 10.1590/1414-431X20187575

**Published:** 2018-04-23

**Authors:** P. Silva, S. Qadir, A. Fernandes, L. Bahamondes, J.F. Peipert

**Affiliations:** 1 Departamento de Tocoginecologia, Faculdade de Ciências Médicas, Universidade Estadual de Campinas, Campinas, SP, Brasil; 2 Department of Obstetrics and Gynecology, School of Medicine, Washington University in St. Louis, St. Louis, MO, USA; 3 Department of Obstetrics and Gynecology, Indiana University School of Medicine, Indianapolis, IN, USA

**Keywords:** Dietary intake, Eating behavior, Body weight, Female, Depot medroxyprogesterone acetate, Contraception

## Abstract

Because of weight gain, women often discontinue hormonal contraception, especially depot medroxyprogesterone acetate (DMPA). Our objective was to conduct a systematic review of studies describing dietary intake or eating behavior in DMPA users to understand whether the use of DMPA is associated with changes in dietary habits and behaviors leading to weight gain. We searched the PubMed, POPLINE, CENTRAL Cochrane, Web of Science, and EMBASE databases for reports published in English between 1980 and 2017 examining dietary intake or eating behavior in healthy women in reproductive age and adolescents using DMPA (150 mg/mL). Of the 749 publications screened, we excluded 742 due to duplicates (96), not addressing the key research question (638), not reporting dietary intake data (4), and not evaluating the relationship of body weight and dietary or eating behaviors (4). We identified seven relevant studies, including one randomized placebo-controlled trial, one non-randomized paired clinical trial, and five cohort studies. The randomized trial found no association and the other reports were inconsistent. Findings varied from no change in dietary intake or eating behavior with DMPA use to increased appetite in the first six months of DMPA use. Few studies report dietary intake and eating behavior in DMPA users and the available data are insufficient to conclude whether DMPA use is associated with changes in dietary habits or behavior leading to weight gain.

## Introduction

One of the most highly effective forms of reversible contraception is depot medroxyprogesterone acetate (DMPA). This progestin-only injectable contraceptive is administered quarterly and works by suppressing ovulation (1). The failure rate of DMPA birth control is 0.3% in the first year of use when it is used correctly (2). However, more than 40% discontinue the method in the first year (3,4), mostly due to weight gain (5,6). Several studies have shown an association between DMPA use and weight and/or fat mass gain in young women (7–10), although the mechanism by which DMPA use causes weight gain is unclear.

There are a number of reports in the medical literature that seek to identify mechanisms to explain weight gain in DMPA users (8 [Bibr B09],^11^–15 [Bibr B14]). Some studies reported changes in dietary intake or eating behavior with DMPA use (7,8,13,16,17). However, after a review of the scientific literature, we did not identify a systematic review addressing the potential association. The goal of this systematic review was to summarize studies that address whether use of DMPA is associated with changes in dietary habits and eating behaviors leading to weight gain.

## Material and Methods

We performed a systematic review of the medical literature on studies reporting dietary intake or eating behaviors and the association with weight gain in DMPA users. Our review followed the guidelines of the PRISMA-P statement (18). We searched for studies that addressed our primary research question: "Can DMPA use change dietary intake or eating behaviors leading to weight gain?"

### Search Strategy

We searched PubMed, POPLINE, Cochrane Central Register of Controlled Trials, SCOPUS, Web of Science, and EMBASE databases for English-language reports published between 1980 and 2017. We used the following search terms: "dietary intake AND contraception", "caloric intake AND contraception", "food intake AND contraception", "dietary intake AND depot medroxyprogesterone acetate", "caloric intake AND depot medroxyprogesterone acetate", "food intake AND depot medroxyprogesterone acetate", "eating behavior AND contraception", and "weight AND depot medroxyprogesterone acetate". We report our search strategies in Appendix S1.

### Inclusion and exclusion criteria

We reviewed publications describing results assessing the association of dietary intake or eating behavior and weight change in healthy women in reproductive-age and adolescents taking DMPA (150 mg, intramuscular injection). We included randomized controlled trials and observational cohort studies (with or without a comparison group), reporting total calorie intake in percentage, kilocalories, or grams, assessed with 24-hour food recall or food frequency questionnaires. In addition, we considered eating behavior as being self-reported changes in appetite by means of yes/no questions or validated questionnaires. We excluded studies that only examined postpartum and breastfeeding women, systematic reviews, short communications, and studies using DMPA as a treatment for specific disorders. We also excluded publications that did not provide data on dietary intake or eating behavior.

### Data collection and analysis

We reviewed all relevant titles and abstracts to determine whether the reports met our inclusion/exclusion criteria. One author (P.S.) extracted the data, entered the information into EndNote¯ reference manager software, and conducted the review for duplicate publications. We reviewed clinical trials and observational studies for potential biases in the study design, blinding, randomization method, number of participants, follow-up, comparison group, outcomes, and attempt to control for confounding factors, following published guidance (19,20).

## Results

**Figure 1.**
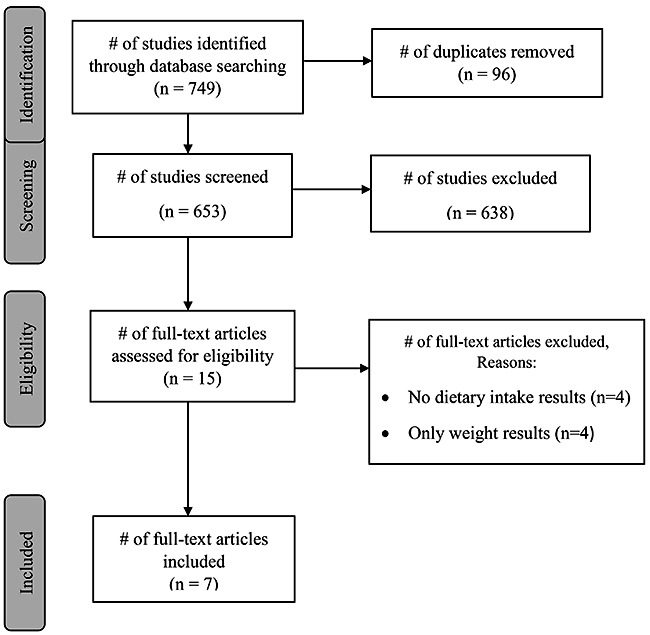
Flow diagram of study inclusion.

We identified 749 publications and excluded 96 articles because they were duplicates and 638 because the titles were not consistent with our primary research question. Additionally, we excluded eight articles because they did not report dietary intake results even though it was described in the methodology. In total, seven studies were eligible for the qualitative analysis (Figure 1). Given the heterogeneity of the studies, we did not perform a quantitative summary or meta-analysis. Supplementary Table S1 contains a qualitative summary of the seven studies (7,8,11,13,16,17,21) included in this systematic review.

In terms of study design, six studies (7,8,11,13,16,21) enrolled U.S. women and one study enrolled Brazilian women taking DMPA (150 mg intramuscular injection) as a contraceptive method (17). One report (11) was a randomized, double-blind, placebo-controlled clinical trial (RCT), one (17) was a non-randomized clinical trial, and the other five (7,8,13,16,21) were observational studies.

### Randomized clinical trial

This trial enrolled 20 healthy adult women between 20 and 35 years of age, with 70% classified as non-Hispanic White, 5% as Asian or Pacific Islander, 10% as Hispanic, and 15% as non-Hispanic black; 95% were nulliparous. The investigators used health-history questionnaires as criteria for eligibility. They excluded women with eating disorders or depression. One group (n=10) received a DMPA injection, and the other group (n=10) received a saline injection. The study assessed body weight, dietary intake, and energy expenditure in different phases of the menstrual cycle. The investigators evaluated diet objectively with a dietary intake scale using three-day records of consumption of food provided by the research team, and energy expenditure by indirect calorimetry. They provided all participants with three meals (breakfast, lunch, and dinner) and choices of snacks and beverages for three days, and investigators recorded how much food the participants consumed. There was no difference between DMPA users and the placebo group regarding body weight (P=0.13), caloric intake (P=0.37), and resting energy expenditure (P=0.76) after the intervention. In both groups, calorie intake (DMPA: 2390 kcal; placebo group: 2245 kcal) was higher than daily energy expenditure (DMPA: 1180 kcal/day; placebo group: 1130 kcal/day). The strengths of this study included the methodology to evaluate dietary intake, a description of the intervention, randomization, and outcomes. Limitations included a small sample size and a short (2 months) follow-up.

### Non-randomized clinical trial

The non-randomized clinical trial (17) was performed with healthy adult Brazilian volunteers. The study assessed dietary intake and body composition in 28 new DMPA users compared to 24 new users of the copper intrauterine device (IUD) matched by body mass index (BMI) and age. Participants were evaluated during 12 months. Two-thirds of the women in both groups were non-white. There was no difference in sociodemographic characteristics, lifestyle habits, and dietary intake between the groups at baseline. However, DMPA users had more years of education than IUD users. To evaluate dietary intake, the researchers used the 3-day food recall method quarterly providing the mean quantities of total energy intake, carbohydrates, protein, and fat. This study assessed body composition by dual energy X-ray absorptiometry reporting the mean of total body fat percentage, total body mass, total lean and fat mass, and central-peripheral fat ratio. The primary outcome of the study was the significant increase of dietary intake in the DMPA group in up to 12 months of use; however, there was no association with weight gain. Differences between the groups included: total energy (P<0.01), carbohydrates (P=0.01), protein (P<0.01), and fat (P=0.01) intake. Regarding body composition, there was an increase in total lean mass (P<0.04) in the DMPA group. The strengths of this study included the methodology, duration of follow-up, and outcomes. Limitations included a small sample size and no randomization.

### Cohort studies

The non-randomized cohort studies (7,8,13,16,21) included in this review enrolled urban healthy women and adolescents between 12 and 33 years of age. Exclusion criteria was eating disorders, contraindication to hormonal contraception, metabolic disease or use of medications that could affect weight, breastfeeding, and the use of DMPA within the past 6 months or use of hormonal contraception (oral contraception, hormonal IUD) within the past 12 months.

All of the observational cohort studies assessed body weight and its association with other variables, including dietary intake and eating behavior, in an attempt to explain reasons for the weight gain in DMPA users. All five studies (7,8,13,16,21) measured body weight objectively and body composition by dual energy X-ray absorptiometry. Four cohort studies (7,8,13,21) evaluated dietary intake by means of 24-hour dietary recall. Two studies (13,21) showed variance measures (SD) of total energy intake and macronutrients (carbohydrates, protein, and fat) intake. One article (16) reported eating behavior by means of a validated questionnaire - the Three Factor Eating Questionnaire - without evaluation of dietary intake. Appetite was evaluated in two studies (7,8) through yes/no questions and scores (16). The follow-up surveys occurred at 6 (16), 12 (13), and 36 months (7,8,21).

Two studies focused on adolescents (13,16) with a mean age of 16.2 years. The participants in the remaining studies (7,8,21) had a mean age of 24.3 years. Two cohort studies (7,21) included a DMPA group and a non-hormonal contraceptive group (methods not specified) as a comparison group. The other three studies (8,13,16) only reported data from DMPA users. The major strength of the cohort studies was the methodology to assess dietary intake (24-hour dietary recall), and the major limitations were small sample size or loss to follow-up.

One prospective study (7) evaluated weight, body fat, and food intake in women between 16 and 33 years old. Groups included users of combined oral contraceptives (COC) (n=245), DMPA (n=240), and non-hormonal contraceptives (n=218). The investigators assessed dietary intake (protein, fat, and carbohydrate) by means of a 24-hour food recall and appetite by means of yes/no questions. After 36 months of follow-up, weight gain (+5.1 kg), body fat (+4.1 kg), and body fat percentage (+3.4%) were higher in DMPA users than in users of COC or non-hormonal methods (P<0.01). Protein intake was protective against weight gain and increase in body fat (P<0.05) among DMPA users. The other dietary variables and appetite were not associated with changes in body weight or body composition in DMPA users. Strengths of this study included a large sample size and a non-hormonal control group. Limitations included the loss to follow-up of more than 24% of DMPA users and missing dietary intake and appetite data (e.g. the number of women that reported change in appetite and the amount of protein and other nutrients consumed).

Another prospective study (8) assessed risk factors for early weight gain (5% gain in the first six months of DMPA use) in 240 women between 16 and 33 years of age (72 non-Hispanic Black, 82 non-Hispanic White, and 86 Hispanic) who used DMPA. The investigators used 24-hour food recall to evaluate quantities of protein, fat, and carbohydrate consumed and they evaluated appetite by means of yes/no questions. Assessments occurred at baseline and every 6 months up to 36 months of use. The investigators noted an association between increased appetite and early weight gain in the first six months of DMPA use (odds ratio 3.1, 95% confidence interval [CI] 1.5–6.2). However, there was no association between calorie intake and weight gain at 12 months. The study had a reasonably large sample size. However, it lacked a non-hormonal comparison group, had a high loss to follow-up (24%), and did not report results regarding macronutrient intake (amount of protein, fat, and carbohydrate consumed).

Le et al. (21) reported a three-year longitudinal follow-up study of 219 DMPA users, 218 COC users, and 171 users of non-hormonal contraceptives (type not specified) between 16 and 33 years old. The researchers evaluated perceived weight gain by yes/no questions, and dietary intake with 24-hour food recall and reported that women who perceived a higher weight gain had a higher caloric intake over time (112 kcal/day, P=0.01). There was no association between DMPA use and change in food intake (P=0.99). The strengths of this study were the follow-up and sample size of DMPA users (more than 200) with hormonal and non-hormonal comparison groups. Retrospective evaluation of dietary intake was the principal limitation of this study.

Lange and colleagues (13) assessed weight, BMI (kg/m^2^), percentage of body fat mass and lean body mass, and dietary intake after one year of DMPA use in adolescents (n=45; mean age=16 years). The researchers evaluated dietary intake by 24-hour food recall; the average caloric intake was 1780 kcal/day (50% from carbohydrates, 35% from fat, and 13% from protein). There was no association of total energy intake (P=0.09) or the consumption of carbohydrates (P=0.15), fat (P=0.08), or protein (P=0.23) with weight gain in DMPA users. However, the consumption of fiber was inversely associated with BMI over time (P<0.05). After one year, the mean BMI and percentage of body fat mass increased, while lean body mass and food intake decreased. Strengths of this report include the methodology to assess dietary intake and report of total energy intake and macronutrients. The major limitations were the small sample size (limited statistical power), lack of body weight data, loss to follow up (31%), and lack of users of a non-hormonal contraceptive method as a comparison group.

The prospective study by Bonny et al. (16) assessed factors associated with weight gain in 43 young new DMPA users, between 12 and 21 years of age. The researchers evaluated weight, BMI, body fat percentage, and lean mass after six months of follow-up. Appetite was measured by the Three Factor Eating Questionnaire. Percent weight and body fat increased (+4.2%, P=0.003 and +12.5%, P<0.001, respectively) in Black adolescents, while appetite decreased overtime. At six months, appetite score was higher in Black adolescents than in White adolescents, but with no association between appetite and weight change in either. Eating restraint (i.e., prevention of eating) and eating disinhibition (i.e. continuation of eating) were predictors of weight gain in Black women. The use of the Three Factor Eating Questionnaire and objective assessment of changes in weight and body composition were strengths of this study. Limitations included small sample size, limited follow-up, users of non-hormonal contraceptive methods as a comparison group, and lack of dietary intake measures.

## Discussion

The articles of this systematic review included two clinical trial reports and five prospective observational studies. Investigators evaluated dietary intake and eating behavior in DMPA users in an attempt to elucidate the possible mechanism of the association between DMPA use and weight variation (7,8,13,16). The RCT found no difference between DMPA users and the placebo group regarding body weight, caloric intake, and resting energy expenditure after the intervention (11). The results of the non-randomized and cohort studies were variable and inconsistent. The non-randomized study showed significant increase of dietary intake in the DMPA group and no association with weight gain (17). Furthermore, one cohort study reported an association between dietary intake and weight gain in DMPA users (21), and three studies reported no association (7,8,13). Eating behavior was a predictor of weight gain in Black DMPA users in one study (16). Two studies showed no association between appetite and weight gain (7,16); however, one of them (8) noted that appetite was predictive of early weight gain after 6 months of DMPA use.

According to data from the US National Survey of Family Growth, 6.5% of women between 15 and 34 years of age chose DMPA as their contraceptive method between 2011 and 2013 (22), with higher rates of DMPA use in younger women (13.9% of women 15 to 19 years of age; 10.1% of 20 to 24-year-old women) (23). Weight variation is a common concern in women, and DMPA users state that weight gain is one of the most common reasons to discontinue use of the method (5).

The mechanism of weight gain in some DMPA users is still uncertain. We performed a systematic review in an attempt to understand whether changes in dietary habits and behaviors are associated with the observed weight gain in DMPA users. The principal strength of the publications was the assessment of dietary intake by 24-hour dietary recall and food frequency records, which are valid and reliable measures to assess food intake in adults (24). Additionally, all the cohort studies assessed weight objectively and used the gold standard technique (25), dual energy X-ray absorptiometry, to evaluate body composition.

The principal limitation of the studies was small sample size (20 women in the RCT, 52 women in the non-randomized study, and fewer than 60 DMPA users at last follow-up in the cohort studies). An average of 35–40% of participants were lost to follow-up in the three studies that reported this measure (7,8,13). One of the main reasons reported in the studies for the loss to follow-up was discontinuation of the method. Other studies have reported similar findings; for example, it was reported (4) that more than 50% discontinued DMPA use in the first year. The US-based CHOICE Project noted that 43% stopped DMPA in the first year (3). This is a major methodological issue with studies addressing consumption and weight change over time with contraceptive use. We included cohort studies in our review even though they had some methodological limitations. The benefits of including observational studies in systematic reviews are the increased generalizability and increased sample size (26,27).

The goal of this review was to assess studies describing dietary intake or eating behavior in DMPA users to elucidate the mechanism of reported weight gain with DMPA use. To the best of our knowledge, there are no other reviews in the literature with this objective. A significant limitation of our review was the inability to perform a quantitative summary or meta-analysis of the data due to methodological differences and heterogeneity of the studies.

Based on our review, we cannot report with confidence that changes in dietary intake and/or eating behavior are the key mechanisms to explain weight gain experienced by DMPA users. Future research should assess dietary intake and eating behavior as well as metabolic parameters and their association with weight change in DMPA users. For women who experience weight gain with DMPA use, careful monitoring of diet and exercise are essential. A healthy lifestyle with a balanced diet that includes consumption of fiber (e.g., fruits and vegetables) and appropriate caloric intake combined with regular physical activity for at least 60 min per day are helpful to achieve and maintain ideal body weight (28–30).

The literature contains few studies reporting dietary intake and eating behavior in DMPA users. The available data are insufficient to conclude that changes in dietary habits or eating behavior are mechanisms leading to weight gain in DMPA users.

## Conflict of interest

J.F. Peipert received research funding from Bayer, Merck, and Teva, and serves on advisory boards for Cooper/Teva and Perrigo.

## Supplementary Material

Click here to view [pdf]
